# Adhesive Gold Foil

**Published:** 1857-06

**Authors:** J. H. M’Quillen


### ADHESIVE GOLD FOIL.

BY J. H. M'QUILLEN.

At the last meeting of the American Dental Convention, when "*The best preparation of Gold for filling Teeth*" was under consideration, the writer stated that "he had tried the plan of annealing foil as proposed by Dr. Arthur, and found that he could not introduce as much gold, when so prepared, into a given cavity as with the ordinary foil received from Abby. He believed the annealed gold hardened under the instrument so rapidly as to choke up the cavity and prevent the perfect consolidation of the filling; and had reason to infer, from his experience, that the specific gravity of a filling of annealed gold is not equal to one of ordinary foil;" of course the same care and force being exercised in the introduction of each.

These remarks were made as the result of casual observations at the time of using the foil, and advanced as impressions rather than asserted as irrefutable facts.

Being desirous of demonstrating to his own satisfaction, whether these views were correct, the writer has, within the last few months, instituted a series of experiments bearing directly upon this point. In pursuing his investigations, he feels indebted to Dr. Buckingham for the use of an ingenious contrivance, made by him some time back, for the purpose of ascertaining the relative amount of crystalline gold and foil that could be introduced into a given cavity. It consists of a steel plate, constructed so as to slide dovetailed into one of brass. The former is perforated with a number of holes, varying in size; the latter serves as a bottom to these.

Having made choice of the cavity to be filled, some No. 5 gold foil of Abby's (as it came from the hands of the manufacturers, with the assurance that it was prepared in the *old-fashioned way*) was torn into half sheets, rolled loosely into ropes, and then each divided into eight or ten pieces by the scissors. The cavity was then filled with the same instruments used in the mouth. After the operation was completed, the plug was forced out of the hole, marked No. 1, and laid aside. A portion of the foil was then prepared according to the directions of Dr. Arthur, and introduced into the *same cavity*, with even greater care and labor than had been bestowed upon the former operation; when finished, it was removed from the plate and marked No. 2. Placing the fillings on the opposite sides of a pair of fine scales, the *ordinary foil*, plug No. 1, was found to be much heavier than the *adhesive foil*, plug No. 2. The smaller weights belonging to the scales had been lost, and it was therefore impossible for the writer to determine the exact difference between them.

Supposing that the disparity of weight in the two fillings might be attributed to his limited experience in the use of gold so prepared; as his only aim and wish was to ascertain the *truth*, and desiring to give the suggestion the most generous margin, the writer called on one of the best operators in the profession, who has been accustomed to use, for the last two years, almost exclusively gold in this form. Having stated the object of his visit, this gentleman very politely consented to fill the same cavity with adhesive gold. After performing the operation with a great deal of care and judgment, (employing at the same time the most delicate instruments in use,) he marked the filling No. 3, when perfected, and placed it with the other two. The three plugs were then weighed carefully in a delicate pair of scales, when the ordinary foil plug, No. 1, was found to weigh ten and three-quarter grains, and Nos. 2 and 3, of the adhesive foil, not over nine and three-quarters each.

If the relative difference, demonstrated by these experiments, should hold good in the operations upon the mouth, when conducted with the care that Dr. Arthur *practices* and *enjoins* on others to observe, it will be found that a cavity which requires ten leaves of No. 5 ordinary foil, or fifty grains, would be filled by nine leaves of No. 5, adhesive foil, or forty-five grains; thus leaving out an entire sheet. Until he obtains more light on this subject, the writer is forced to believe that this ratio of variation would be preserved.

But the difference would be still greater in cases where the operator uses the adhesive gold in large masses; for here the hardening and choking of the gold would prevent, to a more marked degree, the perfect consolidation of the filling. It is asserted by some who use it, that under such circumstances it will not adhere. The writer remembers, however, to have seen an eminent practitioner use it in that way, some months back, without any apparent difficulty in effecting a union between the various particles.

Refraining from further comment, and trusting that others will make this a subject of investigation, the writer would say, in conclusion, that if more carefully conducted experiments should demonstrate the incorrectness of the views he has advanced, no one will rejoice more over such a fact, or more readily acknowledge it, than he will.—*Dental News Letter.*

